# Developing Machine Vision in Tree-Fruit Applications—Fruit Count, Fruit Size and Branch Avoidance in Automated Harvesting

**DOI:** 10.3390/s24175593

**Published:** 2024-08-29

**Authors:** Chiranjivi Neupane, Kerry B. Walsh, Rafael Goulart, Anand Koirala

**Affiliations:** Institute for Future Farming Systems, Central Queensland University, Rockhampton 4701, Australia; chiranjivi.neupane@cqumail.com (C.N.); rgoulartns@gmail.com (R.G.); anand.koirala@cqumail.com (A.K.)

**Keywords:** automation, deep learning, image segmentation, machine vision, mango

## Abstract

Recent developments in affordable depth imaging hardware and the use of 2D Convolutional Neural Networks (CNN) in object detection and segmentation have accelerated the adoption of machine vision in a range of applications, with mainstream models often out-performing previous application-specific architectures. The need for the release of training and test datasets with any work reporting model development is emphasized to enable the re-evaluation of published work. An additional reporting need is the documentation of the performance of the re-training of a given model, quantifying the impact of stochastic processes in training. Three mango orchard applications were considered: the (i) fruit count, (ii) fruit size and (iii) branch avoidance in automated harvesting. All training and test datasets used in this work are available publicly. The mAP ‘coefficient of variation’ (Standard Deviation, SD, divided by mean of predictions using models of repeated trainings × 100) was approximately 0.2% for the fruit detection model and 1 and 2% for the fruit and branch segmentation models, respectively. A YOLOv8m model achieved a mAP50 of 99.3%, outperforming the previous benchmark, the purpose-designed ‘MangoYOLO’, for the application of the real-time detection of mango fruit on images of tree canopies using an edge computing device as a viable use case. YOLOv8 and v9 models outperformed the benchmark MaskR-CNN model in terms of their accuracy and inference time, achieving up to a 98.8% mAP50 on fruit predictions and 66.2% on branches in a leafy canopy. For fruit sizing, the accuracy of YOLOv8m-seg was like that achieved using Mask R-CNN, but the inference time was much shorter, again an enabler for the field adoption of this technology. A branch avoidance algorithm was proposed, where the implementation of this algorithm in real-time on an edge computing device was enabled by the short inference time of a YOLOv8-seg model for branches and fruit. This capability contributes to the development of automated fruit harvesting.

## 1. Introduction

### 1.1. Background

While plant production activities such as broadacre cropping have become highly automated, tree-fruit production has remained highly labor-intensive for activities such as pruning, flower and fruit thinning, harvest forecast through fruit count and sizing and fruit harvest [1]. The automation of these orchard activities requires the use of machine vision. For example, machine-vision-derived fruit lineal measurements can be used for the estimation of the mass of fruit-on-tree based on allometric relationships, as reviewed by [2]. Developments in the use of convolutional neural networks (CNNs), particularly the one-shot detector You Look Only Once (YOLO), and developments in depth camera hardware, e.g., as evaluated by [3], have boosted these applications.

### 1.2. Model Comparisons

Machine vision detection algorithms enable the identification of objects, allowing object count, while a segmentation algorithm also allows for object area estimation in the image. Popular deep learning algorithms used in machine vision include various Region-based Convolutional Neural Network (R-CNN) and YOLO models for localization and Mask R-CNN for segmentation; however, recent versions of the YOLO series have become increasingly used for both purposes [4]. Many publications report on modifications of these mainstream architectures for a particular application; however, the performance of these modifications may be surpassed by improvements in the mainstream models. An ongoing comparative evaluation of the available model architectures is required in the context of specific applications, with the performance typically reported in terms of statistics such as Precision (P), Recall (R), F_1_, and, most commonly, the mean Average Precision (mAP50 and/or the stricter criterion of mAP50-95, which is an average of mAP at IoU 50% to 95% values, in 5% steps).

The performance of a model will be related to its architecture, its training, and the specific use case (test set). The comparison of the performance of models of different architectures, therefore, requires the use of the same training and test sets. It is, therefore, imperative that publications reporting the development of architectures and models for a specific application be accompanied by the release of the training and test data set to enable future evaluations.

Further, repeat trainings of a given CNN model using the same data will not necessarily give the same results when used to predict the same unknowns. Neural networks converge to a local minimum of the loss function, and models can end in different minima depending on the initial conditions and the training process, leading to different predictions [5]. The issue of model performance variation with each training of the model has been sometimes documented in 1D-CNN applications, e.g., [5], but rarely completed in 2D-CNN machine-vision applications. This variation can be due to: (i) floating-point precision with rounding differences accumulating across multiple calculations; (ii) random initialization, if the weights of the neural networks are initialized randomly, leading to different paths during training and resulting in different final models; (iii) stochastic processes in training such as the random shuffling of the training data, stochastic gradient descent (SGD), or other optimization algorithms that include randomness; and (iv) randomness inherent in batch normalization and dropout. If models are trained sufficiently well, however, their performance, as seen in metrics such as mAP, should be similar. Variation in the performance of models resulting from repeat training should thus provide an index to model stability. It is, therefore, sensible to document this variation.

### 1.3. YOLO Comparisons

The YOLO series began in 2015 [6] and has evolved over time, with different versions created by different developers. YOLOv8 is the latest stable model released by Ultralytics (Toluca Hills, CA, USA), who were responsible for YOLOv5 [7], and it was released in January 2023. High accuracy is reported for YOLOv8 in use with the Common Objects in Context (COCO) dataset, e.g., 50.2% mAP50 for the YOLOv8m model [7]. With an improved speed and accuracy compared to its predecessors, YOLOv8 is purported to be the state-of-the-art (SOTA) in both object detection and instance segmentation. The YOLO series has been the topic of multiple recent reviews, e.g., [8,9], which deliver a consistent message that the face-value acceptance of COCO dataset performance values is not appropriate for comparisons, but rather that the models need to be tested in context of an application.

YOLOv8 is based on the PyTorch framework and provides object detection, instance segmentation, image classification and pose estimation functions. Improvements in YOLOv8 include anchor-free detection (reducing the number of prediction boxes whilst speeding up non-maximum suppression), the use of Pseudo Ensembles (i.e., the use of multiple models with different configurations of different hyperparameters during the training process), newer convolutions and the augmentation of images during on-line training (with the model presented with variations of the images used in each epoch, e.g., mosaic augmentation in which four images are stitched together, presenting objects in partial occlusion and against different surroundings). There are five models of different sizes available (nano, small, medium, large and extra-large, referred to as n, s, m, l and x, respectively), varying in the number of model parameters (Table 1). These models provide variation in the speed and accuracy. In general, larger models provide better performance but at reduced inference speed because of the increased computation.

Continuing the YOLO evolution, YOLOv9 was released as an experimental model by Wang, et al. [11] of Ultralytics in February 2024, while YOLOv10 was released by a team of researchers from Tsinghua University in May 2024. Its superior performance to YOLOv8 in object detection has been claimed for both of the new models; however, both are also noted to be more complex and resource-intensive than YOLOv8 for training [12]. The released performance metrics for YOLOv9 do not include inference times (Table 1).

### 1.4. Orchard Application Issues

Three application cases for the use of machine vision in tree-fruit crop production are that of: (i) the count of fruit on-tree, (ii) in-field fruit sizing and (iii) branch avoidance in mechanical harvesting.

#### 1.4.1. In-Orchard Fruit Count

The count of fruit on tree canopies of entire orchards has been accomplished using YOLO localization models [13]. These models allow the count of partly occluded fruit as well as fully exposed fruit; however, a correction factor based on manual counts is still required to correct for the proportion of fully occluded fruit. Some effort has been made to optimize YOLO architectures for such applications, e.g., for mango [14] and citrus [15] fruit counting. However, the rapid improvements made in the main line of the YOLO series may have surpassed the performance of these custom-designed models.

#### 1.4.2. In-Orchard Fruit Sizing

The object detection of fruit in on-tree images is possible even with the object partly occluded, e.g., by leaves or branches, allowing a count of the fruit on the tree. However, fruit sizing is compromised by partial occlusion if fruit dimensions are incorrectly estimated. Geometrical properties such as the diameter of symmetrical fruit can be estimated from a partial outline of the fruit, but this is not possible for non-symmetrical fruit such as mango. Therefore, for partly occluded mango, the fruit must be identified and removed from consideration in an image processing pipeline for fruit sizing.

The measurement of the size of the non-symmetrical (and non-occluded) mango fruit has been reported through the use of image segmentation, rather than bounding box dimensions, working from RGB and depth images of tree canopies [2]. Earlier work [16] involved a manually crafted image segmentation method using image thresholding on the color intensity and morphological operations for the removal of the fruit stalk from the segmented area. An ellipse fitting method was applied to the segmented fruit mask to identify and exclude occluded and improperly segmented fruit from the sizing measurements.

The issues of stalk removal and fruit occlusion by leaves, branches or other fruit were significantly improved by applying a CNN-based instance segmentation method, Mask Regions-CNN (Mask R-CNN) [2]. The recommended method involved the use of an ellipse filter in addition to instance segmentation applied to extracted bounding boxes as the output by a CNN detector. The ellipse filter uses the ratio of the dimensions of major and minor axes of an ellipse fitted to the detected object, with a comparison with the range expected for non-occluded fruit. Fruit sizing was attempted using YOLO4-tiny detection followed by Otsu’s segmentation and Mask R-CNN, with the better result (of an RMSE of 5 mm for the estimation of the fruit length) achieved using Mask R-CNN.

#### 1.4.3. Branch Detection and Segmentation

The automation of tree-fruit pruning and harvesting requires path planning for the mechanical equipment involved in these functions. A core requirement for these applications is the recognition of branches (and other structures) that would impede the equipment. For example, the successful harvest of mango fruit by a Cartesian robot required the placement of the end effector of its arm such that the top fingers of the effector aligned with the top of the fruit. The arm and end effector can safely push through foliage but is impeded by woody branches [17], thus requiring the detection and avoidance of fruit positioned behind larger branches for automated operation.

Branch detection is a more demanding task than fruit detection given the irregular shape of a branch relative to a fruit. This issue necessitates the use of segmentation rather than a bounding box. Several researchers have attempted the trunk and branch segmentation of orchard trees in the context of the automation of the orchard operations of fruit-load manipulation, pruning and harvesting, with the most used imaging system being the Intel RealSense D435 RGB-D camera (Table 2). Of these reports, many involve relatively structured and uncluttered scenes, viz. branches trained to trellis wires and imaging of trees in dormancy, i.e., leafless trees (Table 2). Bounding box detection rather than segmentation has been used for applications involving straight segments (of trunk and branches) [18,19]. A poorer segmentation performance has been reported when attempting to segment smaller and twisted branches, e.g., passionfruit branches [20]. One report [21] compared the use of CNN-based segmentors with a Conditional Generative Adversarial Network (cGAN, Pix2Pix) segmentor [22], noting that the different models provided different advantages. The cGAN provided more information on branch paths, which can be advantageous in reconstructing occluded branch networks.

Mask R-CNN has been the most used algorithm for branch segmentation (Table 2), although a preprint [34] reported a superior performance from a YOLOv8 model (of an unspecified size) than a Mask R-CNN model in terms of both a higher mAP and shorter inference time (YOLOv8 inference time 65% of Mask R-CNN). It is, therefore, timely to benchmark the performance of YOLOv8 for the application of branch segmentation, expanding on the work of [34].

### 1.5. Aims

The current study was undertaken to progress the implementation of machine vision in the tree-fruit applications. The datasets used in this work have been made publicly available to facilitate comparative work by other researchers using the same training and test sets.

CNN models have a stochastic element to their training and thus the performance of models from different trainings can vary, despite the use of the same calibration training and test sets. Despite this, nearly all papers reporting comparisons of model performance are based on a single training. The current study, therefore, undertook to document stochasticity in training in terms of the effect on model performance.

Machine vision can be used in the count and sizing of fruit on-tree, and in-tree-fruit harvesting. Given the rapid improvement in the performance of state-of-the-art models, such as in the YOLO series, it is timely to document the performance of new releases relative to previously developed tailored localization architectures for these applications.

For mango fruit detection and counting, five YOLOv8 and three YOLOv9 models were benchmarked to ‘MangoYOLO’ [14], a bespoke adaptation of YOLOv2. For fruit sizing and harvesting, a segmentation algorithm is required, for which Mask R-CNN provides a benchmark. Specifically, the performance of five YOLOv8 segmentation models of different scales and YOLOv9 (gelan-c-seg) was compared to the benchmark of two Mask R-CNN models, one with a ResNet-50 and the other a ResNet-101 backbones, and to a bounding box-based method [2].

An application case for YOLOv8, the recognition of the relative position of fruit and branches within the ‘picking zone’ of a mechanical harvester [17] to avoid picking an arm interaction with branches, is also explored. This application requires the segmentation of branches in a leaf canopy, a more demanding situation than in a leafless canopy (which is the application case in most literature reports of branch segmentation).

## 2. Materials and Methods

### 2.1. Image Acquisition and Datasets

This study is based on two pre-existing data sets and one newly created dataset (Table 3). At the time of imaging, fruit were past the stone-hardening stage of development, i.e., within four weeks of intended commercial harvest.

**Figure 1:**
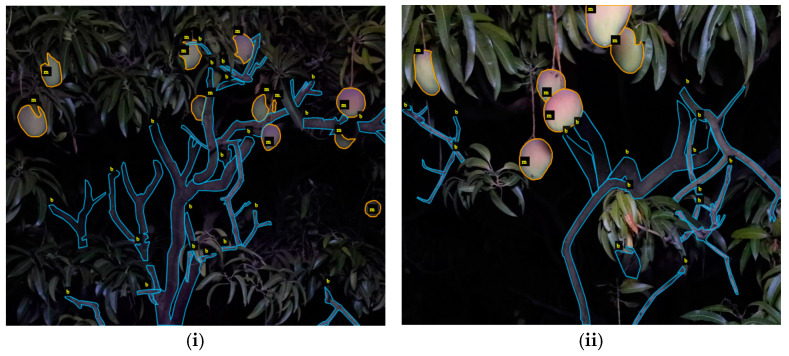
Two examples of ground truth segmentation of visible mango fruit and branches with respective annotations, (**i**) example of fruit occluded by branches and leaves, (**ii**) closer view of fruit occluded by leaves.

Images of all datasets were collected from mango orchards in Central Queensland, Australia. For Dataset-C, an Azure Kinect RGB-D camera (Microsoft, Washington, DC, USA) was mounted in a frame equipped with light-emitting diode (LED) lighting mounted on a vehicle which was driven at about 7 km/h, with images acquired at night, as described in [2,13]. Image tiles of size 640 × 540 pixels were cropped from the original images to create Dataset-C. Images were annotated with ground truth segmentation for branch and fruit classes using the VGG Image Annotator (VIA) [37]. The Polygon annotation tool was used to create two classes (fruit and branches) (Figure 1). Annotations were originally saved in JSON format by VIA, and later converted into COCO style text annotation files. The tile set was allocated on an 80:20 ratio to training and test sets (200 and 50 tiles, respectively).

Mango fruit are convex and smooth, and thus tend to have a high brightness relative to canopy foliage under the artificial illumination used in this study (Figure 1). In contrast, trunk and branches are textured and dark in color, and often heavily shadowed by canopy and fruit. In consequence, the reference (human) labeling of these objects was subject to a greater level of operator interpretation than occurred for fruit labeling.

### 2.2. Model Training

The five versions of YOLOv8 models (n, s, m, l, x) and YOLOv9 (gelan-c) were trained using common settings and hyperparameters for all models. Detection and segmentation models were trained with square input image size of 640 pixels, batch size of 16 with learning rate 0.01, momentum of 0.937 and trained for 100 epochs. Some basic image augmentations were applied such as hue, saturation and value (h,s,v) of 0.015, 0.7 and 0.4, respectively, translate of 0.1, scale factor of 0.5 and flip-left–right of 0.5. For training of Mask R-CNN using Detectron2 [10] platform, the same input image size was used with batch size 8, learning rate 0.001, momentum 0.9 and training for 100 epochs.

A transfer learning approach was adopted using models pre-trained on the COCO dataset, with the number of classes reduced to two for the training of the segmentation models. The models were trained on a high-performance computing system equipped with Intel^®^ Xenon^®^ Gold 6126 CPU 2.6 GHz and Tesla P100 GPU with 16 Gigabytes of graphics memory. Training and testing of the models were conducted using the Ultralytics (https://github.com/ultralytics/ultralytics; accessed: 1 May 2024) Python package. Inference times for prediction of test set images were tested using the hardware used in training and using an edge computing device, Jetson AGX ORIN 32 G (NVIDIA, Santa Clara, CA, USA).

There is a stochastic element to the training of a CNN model which can impact the performance of the resulting model. The default setting in the YOLOv8 has the seed used for random selection of sample batches for training of the model fixed; however, this seed can be manually varied. Ten different seeds were used in training YOLOv8m detection and segmentation models, with mAP (at IoU 50 and 50–95), and these models were used for prediction of the relevant validation set as a means of estimating the impact of this stochasticity on model performance. This was undertaken for both object detection, using the training and test sets of [35], and segmentation, using the training and test sets of [36].

### 2.3. Fruit Count

The training and test datasets (Dataset-A) of [35] were accessed, allowing comparison of the MangoYOLO and YOLOv3 results of that study to the YOLOv8 and v9 models of the current study. Mango detection models were trained using the YOLOv8 small, medium and large architectures. Detection models were used in estimation of count of fruit in test-set images.

### 2.4. Fruit Sizing

The training and test datasets of [36] were accessed, allowing comparison of the Mask R-CNN results of that study to the YOLO models of the current study. Models were trained as described in Section 2.2. Segmentation models were used in estimation of lineal dimensions of fruit in test set images. Pixel height and width of the segmentation mask of fruit were used to derive fruit height and width in real world units (mm) using the distance from camera to fruit as extracted from the associated depth image, following the methodology of [2].

### 2.5. Branch Avoidance in Harvesting

**Figure 2:**
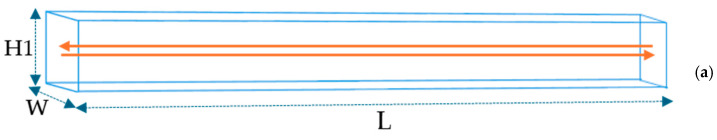

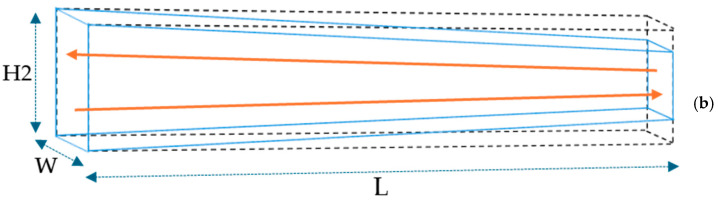
Drawing of a rectangular prism and a side-facing trapezoidal prism, representing the workspace of a harvesting arm moving from home position (left) to destination (fruit) (right), (**a**) without and (**b**) with vertical travel of the arm. L is the distance from the home position to destination (fruit), W is the width of the end effector, H1 is the height of the end effector and H2 is H1 plus the vertical distance travelled by the end effector through the pick cycle.

A use case for YOLOv8 segmentation was developed in context of branch avoidance by an automated mango harvester consisting of a prototype multiple Cartesian robot comprised of a set of horizontal picking arms mounted on a platform that was moved vertically across the face of the tree canopy [17]. Two operation modes of the prototype were considered: (a) the picking arm platform is stationary when the pick cycle is executed and (b) the picking arm platform moves slowly vertically while the pick cycle is executed. In mode (b), vertical movement of the arm during the pick cycle results in a greater vertical area near the home position than at the destination (fruit) position. Thus, the workspace accessed by a harvester arm is a rectangular prism when operated in mode (a) and a trapezoidal prism when operated in mode (b) (Figure 2). The trapezoidal prism consists of two trapezoids joined by four rectangles. The length (L) of the shape is defined by the distance travelled by the arm in a picking event, i.e., distance from gripper palm home position to the fruit, while width (W) is determined by the width of the end-effector. The height (H1) of the rectangular prism in mode (a) is determined by the height of the end effector, while in mode (b), the end planes of the trapezoidal prism have a dimension of H1 plus the distance travelled in vertical movement (Figure 2).

A YOLOv8m-seg model was used for segmentation of fruit and branches. An algorithm was developed in which the segmented point cloud was filtered in the context of the picking arm workspace, i.e., the volume of space accessed by a picking arm during a pick cycle operating in modes (a) and (b), respectively. Three-dimensional point clouds of segmented branches were generated from associated depth image pixels. The presence of branch point cloud within the 3D workspace was then flagged (Figure 2).

In the context of mode (a), a point is inside a rectangular prism defined by 8 vertices if the *x*, *y* and *z* coordinates of the point are within the range of maximum and minimum values of *x*, *y* and *z* coordinates of the rectangular prism vertices. The condition for a point with coordinate *x*, *y* and *z* to be inside a rectangular prism can be defined by:

$$\min\left(x-vertices\right)\leq x\leq \max\left(x-vertices\right)\;\mathrm{AND}$$

$$\min\left(y-vertices\right)\leq y\leq \max\left(y-vertices\right)\;\mathrm{AND}$$

(1) $$z<\max\left(z-vertices\right)$$

where *x*-vertices, *y*-vertices and *z*-vertices represent all *x*, *y* and *z* axis values of vertices, respectively, of a rectangular prism.

For condition (b), when the arms are in continuous vertical movement, the workspace is a trapezoidal prism. A trapezoidal prism is defined by 8 vertices and 6 planes. To identify if a portion of a branch is present within this workspace, each point of a branch point cloud can be tested for occurrence inside each face of the trapezoidal prism, i.e., each point can be classified as lying inside and outside of each plane of the trapezoidal prism (Figure 2).

The location of a point in a 3D coordinate system can be determined with respect to a 3D plane by fitting the point to the equation of each face of the trapezoidal prism and matching the sign of the result with pre-defined rule. Consider the equation of a plane defined by three points in a 3D Cartesian coordinate system using point-normal form of the equation of a plane. Three vertices of a trapezoidal prism, $A(x_{1},\;y_{1},\;z_{1})$, $B(x_{2},\;y_{2},\;z_{2})$ and $C(x_{3},\;y_{3},\;z_{3})$, and the vectors $\vec{AB}\;$(Equation (2)) and $\vec{AC}\;$(Equation (3)) lie on one face (plane) of the trapezoid.

(2) $$\vec{AB}=B-A=\left(x_{2}-x_{1}\right),\left(y_{2}-y_{1}\right),(z_{2}-z_{1})$$

(3) $$\vec{AC}=C-A=\left(x_{3}-x_{1}\right),\left(y_{3}-y_{1}\right),(z_{3}-z_{1})$$

The orthogonal vector (normal) $\vec{n}$ perpendicular to this plane is obtained by the cross product of positional vectors $\vec{AB}\times \vec{AC}$ (Equation (4)).

(4) $$\vec{n}=\vec{AB}\times \vec{AC}=\left|\begin{array}{ccc} \vec{i} & \vec{j} & \vec{k}\\ \left(x_{2}-x_{1}\right) & \left(y_{2}-y_{1}\right) & \left(z_{2}-z_{1}\right)\\ \left(x_{3}-x_{1}\right) & \left(y_{3}-y_{1}\right) & \left(z_{3}-z_{1}\right) \end{array}\right|$$

This calculation reveals coefficients of $\vec{i}$, $\vec{j}$ and $\vec{k}$ as (a, b, c). The (a, b, c) coefficients from a normal vector of a plane can be used to obtain the value of d for each point in the point cloud by solving the scaler equation of a plane ax+by+c+d=0. A point on a plane will satisfy the equation. The point position relative to the side of the plane is indicated by a positive or negative (parity) value of the result obtained from the equation.

A potential limitation of this approach in field harvesting is a high compute time. It is desired to process frames at 25 fps on a Jetson AGX Orin platform in the harvesting application, thus there is <40 ms per frame available for processing. The time for fruit and branch segmentation, 3D point cloud generation and filtering of the cloud in context of either a rectangular, a trapezoidal or both spaces was measured using Jetson AGX Orin hardware.

## 3. Results and Discussion

### 3.1. Fruit Detection

By default, the training of YOLOv8 models involves the use of a fixed seed value for the random selection of training data batches. The repeated training of a YOLOv8 model (with default settings) produced models that were identical in the test set performance. This result indicates that the impact of rounding associated with the floating-point value was negligible to model development.

To quantify the effect of variation in the sample order, a YOLOv8m model was trained and tested 10 times using the same mango dataset (Dataset-A) [35], with variation in the randomness seed used in the model. The mAP50 ‘coefficient of variation’ (Standard Deviation, SD, divided by mean of predictions using models of repeated trainings × 100) was approximately 0.2% for the fruit detection model, while that for the stricter criterion of mAP50-95 was 0.36% (Table 4). This estimate of variation provides a guide for judging the significance of differences in mAP obtained with different models.

The YOLOv8s and m models outperformed the benchmark MangoYOLO and YOLOv3 in terms of both the mAP and inference time (Table 5). Given the stochasticity of the model training, there was no effective difference in the mAP50 achieved by the YOLOv7 and v8 variants (which varied by only 0.2 units).

### 3.2. Fruit and Branch Segmentation

The performance of YOLOv8m-seg models resulting from the 10 trainings with variation in the seed used for randomness used in the training process was evaluated using Dataset-C (Table 3). The mAP50 ‘coefficient of variation’ was approximately 1 and 2% for fruit and branch segmentation models, respectively (Table 6). The higher training variation for the branch than the fruit model is consistent with the branch model being poorer (lower accuracy and robustness) than the fruit model.

**Figure 3:**
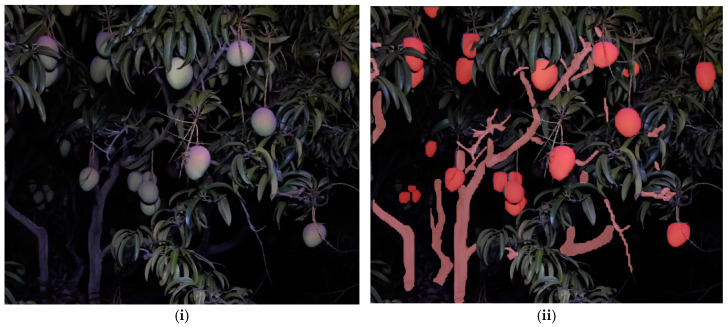
Example of fruit and branch segmentation using YOLOv8m segmentation model. (**i**) Original RGB image, (**ii**) segmented image.

Two Mask R-CNN models, with ResNet-50 and ResNet-101 backbones, respectively, and five YOLOv8 segmentation models (n, s, m, l, x) were trained for localization of fruit and branches (with example output shown in Figure 3).

Models trained using fruit data only performed slightly better than models trained with both fruit and branch data in terms of the segmentation of fruit (Table 7) and branches (Table 8). For example, the YOLOv8s-seg model achieved a mAP50 of 98.7 in the localization of fruit when trained on fruit data only and 97.9 when trained on fruit and branch data. The performance of YOLOv9 (gelan-c-seg) was similar to YOLOv8l-seg in terms of P, R and mAP50 and to YOLOv9-m-seg in terms of the inference time.

The Mask R-CNN (ResNet-101) models performed slightly better than the Mask R-CNN (ResNet-50) models, except for the fruit-only dataset where the results were comparable; however, both were outperformed by the YOLO models (Table 7 and Table 8). For example, the YOLOv8m-seg model achieved a mAP50 of 80.5 in the localization of fruit and branches, compared to 67.2 for the Mask R-CNN (R101) model. The increase in performance of the YOLO models over the Mask R-CNN models was accentuated for the branch localization task, compared to the fruit localization task (Table 7 and Table 8). All YOLO models, including the x model, ran with shorter inference times than the Mask R-CNN models.

There was little effective difference in the performance between the s, m, l or x YOLOv8-seg and YOLOv9 (gelan-c-seg) models in terms of the mAP or F_1_ score for either fruit (Table 7) or branch (Table 8) localization. For example, the highest mAP for fruit segmentation was achieved with a YOLOv8s model; however, the maximum difference across the v8 models was only 0.6 units (Table 6), less than the variation seen in the re-training of a model (0.9, Table 7).

As expected, the inference time increased with the model size (e.g., four-fold between the n and x models) (Table 7 and Table 8). As expected, the inference time was also dependent on the computing hardware and software (Table 9).

The choice of a model involves a balance between the model accuracy and inference speed in context of the application needs. Speed is hardware-dependent, while proprietary software development kits (SDK) such as NVIDIA^®^ TensorRT™ optimize inference and provide a runtime with a low latency and high throughput (Table 9). However, the limitation of TensorRT is that it is tied to the use of modern NVIDIA hardware. Note also that the model conversion to TensorRT (FP-16) may result in a small loss in accuracy.

It is desired to process depth images in real-time at around 25 frames per second for a fruit harvesting application involving a continuous vertical movement of harvesting arms, i.e., a maximum inference time of <40 ms. Longer inference times are acceptable in the fruit size distribution application, where images are sampled at < 1 image per 2 s (for a driving speed of 7 km/h) or may be post-processed, after imaging is completed [2]. For Platform A, a desktop system specification relevant to post-processing or point cloud processing, the <40 ms requirement was achieved using all TensorRT, FP-16 YOLOv8 models and all but the extra-large PyTorch YOLOv8 models (Table 9). For Platform B, a low-powered edge computing platform specification relevant to deployment in orchard equipment, the criterion was achieved using medium and smaller models using PyTorchYOLOv8 models.

### 3.3. Fruit Sizing

The estimation of the fruit lineal dimension from a depth image was improved (i.e., RMSE decreased) with the use of Mask R-CNN segmentation over a bounding box-based method, as previously reported [2]. Further improvement was not achieved with the use of YOLOv8 models (Table 10). Mask R-CNN outperformed the YOLO models in terms of bias values on the estimation of the fruit length for cultivar Keitt but not Honey Gold. The cause of this failure is not clear.

### 3.4. Branch Localization and Avoidance

**Figure 4:**
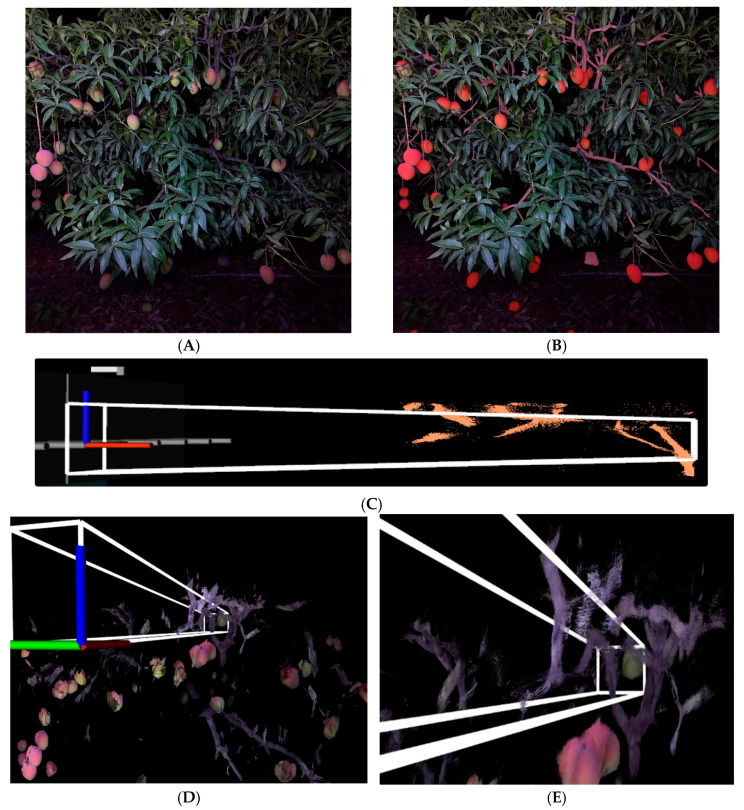
(**A**) Raw RGB image; (**B**) segmented fruit and branch in 2D image; (**C**) side view of segmented 3D point cloud with projected harvester workspace and rectangular prism filtering; and (**D**,**E**) close-up of 3D point cloud showing target fruit with workspace containing branches.

In the mechanical harvest approach described by [17], the trapezoidal prism shaped workspace of each picking cycle should be free of obstacles, defined as branch segments in the current application. This was estimated by calculating the position of each point of the YOLOv8m segmented branch point cloud (Figure 4B) in the context of inclusion within the picking workspace (Figure 4C–E).

While theoretically sound as an approach to avoid harvester arm interaction with branches, a practical consideration is the computing time. The YOLOv8m-seg model is attractive for this application given its low inference time on an edge computing device (Table 9). The time to filter the point cloud for points associated with branches was measured for the rectangular prism expanded-arm workspace and the actual trapezoidal arm workspace, using either CPU or GPU resources of the Jeston AGX Orin (Table 11). As expected, the filtering time was reduced with the use of GPU resources. Filtering to a rectangular prism was faster than for a trapezoidal prism due to the simpler calculations involved. The filtering time of all approaches was well within that required for processing data in real-time at 25 fps for the harvest of a single fruit. However, the Cartesian robot design allows for multiple arms, with the simultaneous harvest of multiple fruit. This requirement favors the use of the rectangular prism in filtering, and the use of CUDA.

## 4. Conclusions

Stochasticity in model training was observed, although for the models tested, the variation in the performance was small with a 0.2% coefficient of variation for fruit detection and 1 and 2% for fruit and branch segmentation using the YOLOv8m model. Nonetheless, it is recommended that this variation be documented in studies undertaking the comparison of models.

The performance of YOLOv8 models was documented in the context of three tree-fruit applications, the (i) fruit count, (ii) fruit sizing and (iii) branch avoidance in automated harvest. The YOLOv8m model is recommended for outperforming the purpose designed MangoYOLO for the application of the detection and count of mango fruit on images of tree canopies, with real-time detection using an edge computing device as a viable use case. For fruit sizing, the accuracy of YOLOv8m-seg was similar to that achieved using Mask R-CNN, but the inference time was much shorter, again an enabler for the field adoption of this technology. The short inference time (allowing >25 fps) of a YOLOv8-seg model for branches enabled its application in a branch avoidance algorithm, contributing to the development of automated fruit harvesting. For branch avoidance, a filtering time of <2.1 ms was achieved for various point cloud filtering methods using a GPU-enabled edge computing device.

The continued benchmarking of new model architectures for a given application using the same training and test sets is recommended. In particular, the remarkable advance of the YOLO series will see continued application development and we anticipate the further evaluation of a stable release version of YOLO v9 and YOLOv10 using the datasets used in the current study (which are publicly available as detailed in the Data Availability section below). We advocate for the release of training and test datasets with any work reporting model development and for the documentation of the performance of the re-training of a given model, quantifying the impact of stochastic processes in training.

## Figures and Tables

**Table 1:** CNN model architecture of the YOLOv8 series segmentation and detection models and the benchmark Mask R-CNN segmentation model, described in terms of floating point operations in billion (BFLOPs) and performance attributes in context of the COCO data challenge (data sourced from [7,10,11]).

	Instance Segmentation			Detection		
Model	Speed (ms)	mAP (COCO)	BFLOPS	Speed (ms)	mAP (COCO)	BFLOPs
Mask R-CNN (ResNet-101-FPN)	195 (Tesla M40 GPU)	35.7	347.6	195	38.2	-
YOLOv8n	96.1 (ONNX CPU)	30.5	12.6	80.4(ONNX CPU)	37.3	8.7
YOLOv8s	155.7 (ONNX CPU)	36.8	42.6	128.4(ONNX CPU)	44.9	28.6
YOLOv8m	317.0 (ONNX CPU)	40.8	110.2	234.7(ONNX CPU)	50.2	78.9
YOLOv8l	572.4 (ONNX CPU)	42.6	220.5	375.2(ONNX CPU)	52.9	165.2
YOLOv8x	712.1 (ONNX CPU)	43.4	344.1	479.1(ONNX CPU)	53.9	257.8
YOLOv9-T	-	-	-	-	38.3	7.7
YOLOv9-S	-	-	-	-	46.8	26.4
YOLOv9-M	-	-	-	-	51.4	76.3
YOLOv9-E	-	-	-	-	55.6	189.0
YOLOv9-c	-	43.5	145.5	-	53.0	102.1
YOLOv9 (gelan-c)	-	42.4	144.6	-	52.3	102.1

Note: ‘-’ indicates data are not available for COCO evaluation from official software repository, ms—milliseconds.

**Table 2:** Summary of reports of tree-fruit branch segmentation.

Crop/Context	Camera	Segmentation Method	Statistics	Reference
apple leafy and leafless tree trunk detection of trees with branches trained to trellis wire	Point Grey Ladybug3 360-degree camera	R-CNN	87–96% and 99% accuracy in leafy and leafless trees, respectively	[23]
apple leafless tree branch segmentation for automated tree training systems	Kinect v2	SegNet (semantic segmentation)	0.92 for trunk and 0.93 for branch mean IoU of 0.59 and 0.49 and a Boundary F_1_ score 0.03 and 0.88	[24]
rose leafless branch 3D reconstruction for pruning	stereo pair cameras with color and grayscale image pair acquired (camera detail not given)	Fully Convolutional Segmentation Network (adaptation of Unet and ResNet architecture)	branch detection accuracy of 88% with pixel level segmentation F_1_—91.06%	[25]
litchi leafy branch detection for harvesting	Nikon D5300 (RGB only)	UNet segmentation within YOLOv3 bbox ROI	P 0.95 in high light; 0.96 in normal and low light MIoU 79.0, 84.3 and 78.6 under high, medium and low light, respectively	[26]
apple leafy branches trained to trellis wire for catch and shake harvesting	RealSense d435i	Faster R-CNN	mAP of 82.4%	[27]
jujube leafless tree for pruning	Azure Kinect DK	SPGnet	accuracy for trunk 93%; branches 84%	[28]
apple leafy tree branches for tree pruning and fruit thinning		CNNS: U-Net and DeepLabv3 Conditional GAN: Pix2Pix	Binary accuracy P2P 97.3%, U-Net 97.8%, DeepLabv3 98.0%	[21]
citrus for fruit harvesting	Kinect v2	Mask R-CNN	Avg. P 96.3%	[29]
cherry leafless tree pruning	RGB (detail not given)	optical flow network (FlowNet2) with GAN Pix2Pix	IOU < 70%, FP < 3%, FN < 33%	[30]
cherry leafless tree pruning	2 stereo cameras (detail not given)	3D point cloud classification as branch using a CNN (detail not given)	accuracy of 70%	[31]
apple leafy tree fruit and trunk detection for fruit tracking	Microsoft Kinect V2	YOLOv4-tiny	detection mAP 99.35%	[32]
apple leafless branch segmentation for robotic pruning	Nikon D750 (RGB only)	Mask R-CNN, Cascade Mask R-CNN with ResNet50 and Swin Transformer	mAP for bbox 0.943 and segmentation 0.940, using an Intersection over Union (IoU) of 0.5	[33]
pomegranate leafy branch reconstruction for robotic harvesting	RealSense D435	modified YOLOv4 bounding box and color-based thresholding with angle constraints on identified branches, branch reconstruction	P 90.7%, R 89%, F_1_ 90%	[19]
apple leafless and leafy tree trunk detection	unnamed RGB only	YOLOv5s bbox with K-means clustering to calculate anchor frame; with Squeeze-and-Excitation module	mAP 95.6, 98.4, 96.5 and 89.6% in spring, summer, autumn and winter	[18]
passionfruit leafy branches for harvesting and phenotyping	RealSense D435	Mask R-CNN	average precision, average recall and F_1_ scores 0.64, 0.77 and 0.70, respectively	[20]
apple leafless and leafy trees for harvesting and crop load manipulation	RealSense 435i	Mask R-CNN YOLOv8-seg	leafless trees mAP: Mask R-CNN—82.8% (trunk), 67.3% (branch) YOLOv8—97.1% (trunk), 91.9% (branch)	[34]

**Table 3:** Image datasets used in this study.

Dataset	Details
Dataset-A	MangoYOLO dataset of RGB images of mango canopies with ground truth of labeled bounding boxes on fruit, from [35]
Dataset-B	Mango instance segmentation dataset of RGB images of mango canopies with ground truth of segmented fruit, from [36]
Dataset-C	Mango-branch (2 classes) instance segmentation dataset of RGB images of mango canopies with ground truth of segmented fruit and branches. Dataset created for this study and publicly available from https://doi.org/10.25946/26212598.v1 (accessed on 5 July 2024)

**Table 4:** Stochasticity in YOLOv8m model training—variation in test set Precision, Recall and mAP for 10 models trained using the same training set but different model seeds.

Metrics	Mean (%)	SD (%)	Min–Max (%)
P	96.21	0.90	4.1
R	94.56	0.92	2.8
mAP50	98.45	0.21	0.7
mAP50-95	77.64	0.28	1.0

**Table 5:** Comparison of YOLOv8 detection models to MangoYOLO and YOLOv3 models for localizations of mango fruit in tree canopy images, with the use of the same training and test set images for all models. Inference time per image is averaged across the 130 test images. Best value for a given statistic is shown in bold. ‘-’ indicates results not reported in [14].

Model	mAP @IoU = 0.5	mAP @IoU = 0.5–0.95	Inference (ms)
MangoYOLO ^1^	98.55	-	15
YOLO v2 ^1^	95.90	-	20
YOLO v3 ^1^	96.70	-	25
YOLOv3-tiny	98.06	62.8	13.5
YOLOv4	99.20	70.9	40.2
YOLOv4-tiny	98.63	68.0	14.1
YOLOv7	99.11	71.4	38.6
YOLOv7-tiny	99.02	70.6	18.6
YOLO v8s	**99.31**	76.9	**7.7**
YOLO v8m	99.29	**77.6**	14.1
YOLO v8l	99.26	76.9	23.6
YOLOv9-c	99.30	76.9	21.6
YOLOv9-e	99.20	77.1	43.9

^1^ Results from [14].

**Table 6:** Test set mean, SD and minimum and maximum mAP50 values from 10 repeated trainings of a YOLOv8m segmentation model, with different seed values for random selection of training data batches used in each model training.

Class	Metrices	Mean	SD	Max-Min
Overall	P	79.92	0.93	3.1
	R	74.74	1.29	4.1
	mAP-50	78.78	0.56	1.8
Branch	P	65.74	1.87	6.3
	R	53.68	2.07	7.1
	mAP-50	59.24	1.06	3.5
Fruit	P	94.1	1.00	3.5
	R	95.79	0.73	2.2
	mAP-50	98.33	0.24	0.9

**Table 7:** Mask R-CNN and YOLOv8 segmentation model performance for fruit detection in a test set of images for models trained as either fruit-only or fruit and branch segmentors. Best value for a given statistic is shown in bold.

Dataset	Model	P (%)	R (%)	mAP50 (%)	mAP50-95 (%)	F_1_ Score (%)	Inference Time (ms)
fruit and branch (Dataset-C)	Mask R-CNN (R-101) All fruit	-	-	67.8 95.6	50.1 83.8	-	62.3
	Mask R-CNN (R-50) All fruit	-	-	64.68 94.5	49.7 **84.9**	-	48.4
	YOLOv8n-seg All fruit	80.2 94.2	75 **96.0**	78.1 97.8	53.9 83.8	77.5 95.1	**11.9**
	YOLOv8s-seg All fruit	82.3 **96.5**	76.0 96.0	79.7 97.9	56.1 84.8	79.0 **96.2**	14.0
	YOLOv8m-seg All fruit	78.8 90.7	77.6 **96.0**	80.5 97.7	57.3 83.8	78.2 93.3	21.6
	YOLOv8l-seg All fruit	80.4 95.5	75.4 94.4	79.4 96.9	56.7 84.6	77.8 94.9	30.6
	YOLOv8x-seg All fruit	76.6 90.9	76.0 96.0	78.1 97.5	56.4 84.5	76.3 93.4	42.2
	YOLOv9 (gelan-c-seg) All fruit	80.8 93.6	76.7 **96.3**	81.1 **98.3**	57.2 84.2	78.7 94.9	23.2
fruit only (Dataset-C)	Mask R-CNN (R-101)	-	-	97.0	**85.2**	-	60.0
	Mask R-CNN (R-50)	-	-	97.4	85.5	-	51.1
	YOLOv8n-seg	96.3	93.7	98.1	82.2	95.0	**3.6**
	YOLOv8s-seg	**97.7**	**94.7**	**98.7**	84.2	**96.2**	9.1
	YOLOv8m-seg	97.6	94.4	98.4	84.2	96.0	13.4
	YOLOv8l-seg	95.3	94.5	**98.7**	85.6	94.9	21.3
	YOLOv8x-seg	96.6	94.2	98.2	85.1	95.4	31.3

Note: Precision, recall and F_1_ values for Mask R-CNN models are not produced by Detectron2 implementation.

**Table 8:** Mask R-CNN and YOLOv8 segmentation model performance for fruit detection in a test set of images for models trained as either branch-only or fruit and branch segmentors. The best result for a given statistic is shown in bold.

Dataset	Model	P (%)	R (%)	mAP50 @0.5 IoU (%)	mAP50-95 (%)	F_1_ Score (%)	Inference Time (ms)
fruit and branch (Dataset-C)	Mask R-CNN (R-101) All branch	-	-	67.2 40.0	50.1 16.4	-	62.3
	Mask R-CNN (R-50) All branch	-	-	64.7 34.9	49.7 14.5	-	48.4
	YOLOv8n-seg All branch	80.2 66.3	75.0 53.9	78.1 58.4	53.9 25.0	77.5 59.5	**11.9**
	YOLOv8s-seg All branch	**82.3** 68.2	76.0 56.0	79.7 61.5	56.1 27.5	**79.0** 61.5	14.0
	YOLOv8m-seg All branch	78.8 66.9	**77.6** 59.3	**80.5** **63.2**	57.3 **30.7**	78.2 62.9	21.6
	YOLOv8l-seg All branch	80.4 65.3	75.4 56.4	79.4 61.9	56.7 28.8	77.8 60.5	30.6
	YOLOv8x-seg All branch	76.6 62.3	76.0 55.9	78.1 58.6	56.4 28.3	76.3 58.9	42.2
	YOLOv9 (gelan-c-seg) All branch	80.8 67.9	76.7 57.1	81.1 63.9	57.2 30.2	78.7 62.0	23.2
branch only (Dataset-C)	Mask R-CNN (R-101)	-	-	46.08	17.2	-	50
	Mask R-CNN (R-50)	-	-	41.14	16.9	-	49
	YOLOv8n-seg	60.3	55.9	56.4	25.6	58.0	3.2
	YOLOv8s-seg	**61.6**	59.8	59.7	27.5	60.7	9.1
	YOLOv8m-seg	60.9	**63.0**	60.4	**29.8**	**61.9**	12.5
	YOLOv8l-seg	60.1	61.7	**60.9**	29.4	60.9	21.6
	YOLOv8x-seg	59.6	60.3	58.5	28.0	59.9	31.2

Note: Precision, recall and F_1_ values for Mask R-CNN models are not produced by Detectron2 implementation.

**Table 9:** Inference speed of the YOLOv8-seg models for an image input size of 640 × 640 pixels. Platform A—a desktop resource: Intel Xeon 2.5 GHz with Tesla P100 16 GB GPU, Platform B—an edge computing resource: NVIDIA Jetson AGX ORIN 32 GB. Abbreviations: GFLOPS—Giga Floating Point Operations per Seconds, TRT—Tensor RT model. Percentages in brackets are values relative to the values for Platform A (PyTorch).

Model	GFLOPs	Weight File Size (MB)	Inference Time (ms)		
			Platform A (PyTorch)	Platform B (PyTorch)	Platform A (TensorRT, FP-16 Model)
MaskR-CNN(R101)	290	242	50	-	-
YOLOv8n-seg	12.0	6.4	3.3	16.5 (500%)	2.8 (85%)
YOLOv8s-seg	42.4	22.7	7.2	22.5 (313%)	4.9 (68%)
YOLOv8m-seg	110.0	52.3	18.5	31.1 (168%)	9.2 (50%)
YOLOv8l-seg	220.1	88.0	30.0	40.3 (134%)	14.6 (49%)
YOLOv8x-seg	343.7	137.2	50.1	55.2 (110%)	19.8 (40%)

**Table 10:** Prediction statistics for estimation of fruit length (L, in mm).

Cultivar	Honey Gold (*n* = 38)			Keitt (*n* = 26)		
Segmentation Model	Bias	RMSE	RMSE-bc	Bias	RMSE	RMSE-bc
YOLOv4-bbox + Otsu ^1^	3.0	5.9	-	−2.1	6.8	-
Mask R-CNN (ResNet-101) ^1^	3.1	4.7	-	2.4	5.1	-
YOLOv8s-seg	1.2	4.8	4.6	4.8	6.7	4.7
YOLOv8m-seg	1.3	4.4	4.2	6.4	7.5	3.6
YOLOv8l-seg	1.9	4.6	4.2	6.4	7.1	3.3

RMSE—root mean square error, bc—bias corrected, ^1^ Result from [2].

**Table 11:** Time taken to filter segmented point cloud to identify branch obstacles within a harvester arm workspace defined as a rectangular prism or a trapezoidal prism. The point cloud consisted of ~4.2 million points.

Filtering Method	Filtering Time (ms)	
	CPU	CUDA
Rectangular prism only	5.91	1.33
Trapezoidal prism only	10.3	2.1
Trapezoidal (after rectangular prism)	0.65	0.27
Rectangular prism + trapezoid prism	6.56	1.63

## Data Availability

Parts of the image dataset created and used for the training of deep-learning models in this study are published and available at https://doi.org/10.25946/26212598.v1 (accessed on 17 July 2024).
